# Preparing Future Surgeons: An Evaluation of Academic Surgeons' Views on Laparoscopic Simulation Training for Medical Students

**DOI:** 10.7759/cureus.37924

**Published:** 2023-04-21

**Authors:** Joshua D Collingwood, Cole S Arnold, Benjamin L Crews, Alina F Faunce, Britton A Ethridge, Natalie Barefield, Pankaj Dangle, Sherry L Roach

**Affiliations:** 1 Department of Research, Alabama College of Osteopathic Medicine, Dothan, USA; 2 Department of Primary Clinical Skills, Alabama College of Osteopathic Medicine, Dothan, USA; 3 Pediatric Urology, Indiana University Health, Indianapolis, USA

**Keywords:** surgical skills-based training, basic surgical skills, laparoscopic surgery, undergraduate medical education, laparoscopic simulation

## Abstract

Introduction

With the development of laparoscopic simulation, medical students are motivated to expand their knowledge and proficiency in basic surgical skills. This study aims to demonstrate their capability and readiness for surgical clerkships and, ultimately, surgical residency. This study’s primary objective is to ascertain academic surgeons’ perspectives regarding laparoscopic simulation in undergraduate medical education and to determine if early exposure may afford medical students additional opportunities during their surgical clerkships.

Methods

A survey was created to ascertain surgeon perspectives on medical students’ early exposure to laparoscopic simulation. Five-point Likert scales were used to indicate surgeon perspectives. The survey was conducted over the two days of the meeting; all attendees' meeting inclusion criteria were encouraged to participate. Surgeons practicing within the state of Alabama, with prior experience overseeing the development and training of medical students before June 1, 2022, and attending the AL Chapter American College of Surgeons 2022 Annual Meeting were eligible to complete the survey. Only completed surveys were included for analysis.

Statement 1: “Pre-clinical exposure to laparoscopic simulators is beneficial to the training and development of medical students pursuing a surgical career.”

Statement 2: “I am more likely to allow medical students to participate in laparoscopic surgery cases if they have had prior exposure to, and training with, laparoscopic simulators.”

Results

An on-site survey was conducted among 18 surgeons consisting of 14 full-time faculty attendings, two post-graduate year-five residents, and two post-graduate year-three residents, all practicing in academic medicine with experience overseeing the training of medical students. In response to Statement 1, 33.3% of respondents strongly agree and 66.6% agree. In response to Statement 2, 61.1% of respondents strongly agree, 33.3% agree, and 5.6% were undecided.

Discussion

Our study provides evidence to support the inclusion of laparoscopic simulation training in undergraduate medical education to enhance medical students' basic surgical skills and improve their clinical experience. Further research could inform the development of effective laparoscopic simulation training programs that prepare medical students transitioning to surgical residency.

## Introduction

Laparoscopy has revolutionized the field of surgery over recent decades, becoming the standard of care for many procedures across various specialties [1]. The shift toward minimally invasive techniques has significantly reduced the risk of complications, pain, and recovery time compared to open surgery [2]. Despite these benefits, laparoscopy has significant challenges in both learning and teaching. The learning curve is steep, and the complex skills needed to perform laparoscopic procedures effectively require considerable training and experience [3]. The loss of depth perception, limitations to visuospatial awareness, lack of direct feedback from tissues, and the fulcrum effect of the instruments all contribute to the difficulty in mastering laparoscopy [4]. Teaching laparoscopic surgery also presents its own set of challenges [5]. The surgeon has limited direct control over trainee actions. To address these challenges, laparoscopic simulation has emerged as an effective solution. Laparoscopic simulation offers a safe and effective way for surgical trainees to develop and refine the skills necessary to perform complex surgical procedures in a controlled environment. The laparoscopic simulation also addresses the limitations of direct control and standardization in teaching methods. Evidence supports that simulation-based training is an effective method for developing and translating laparoscopic skills to the operating room, with fewer errors, shorter operation times, and increased patient safety [6-8].

With the development of laparoscopic simulation, medical students are motivated to expand their knowledge and proficiency in basic surgical skills. This aims to demonstrate their capability and readiness for surgical clerkships and, ultimately, surgical residency. This study’s primary objective is to ascertain academic surgeons’ perspectives regarding laparoscopic simulation in undergraduate medical education and to determine if early exposure may afford medical students additional opportunities during their surgical clerkships.

## Materials and methods

The Alabama College of Osteopathic Medicine Institutional Review Board deemed this study exempt. Participation in the anonymous survey was voluntary for all surgeons, and the survey results were summarized without revealing any personal identification.

Survey population

Surgeons practicing within the state of Alabama, with prior experience overseeing the development and training of medical students before June 1, 2022, and attending the AL Chapter American College of Surgeons 2022 Annual Meeting were eligible to complete the survey.

Survey and implementation

**Figure 1:**
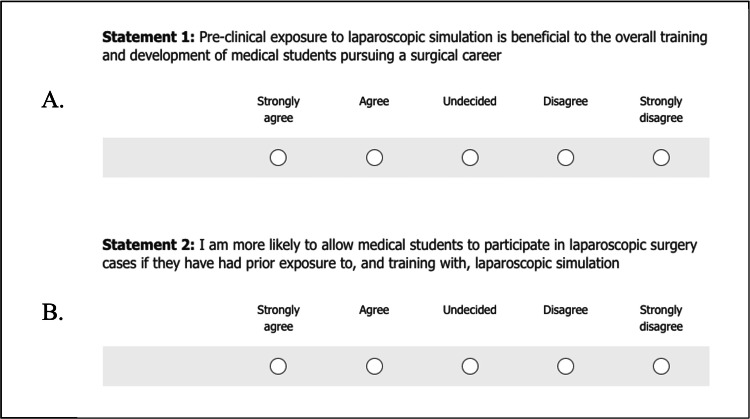
Survey statements assessed using 5-point Likert scales to ascertain academic surgeons’ perspectives regarding (A) laparoscopic simulation in undergraduate medical education and (B) if early exposure may afford medical students' additional opportunities during their surgical clerkship.

A survey was created to ascertain surgeons' perspectives on medical students’ early exposure to laparoscopic simulation. Five-point Likert scales were used to indicate surgeons' perspectives (Figure. 1). The survey was conducted over the two days of the meeting; all attendees' meeting inclusion criteria were encouraged to participate. Only completed surveys were included for analysis.

## Results

**Figure 2:**
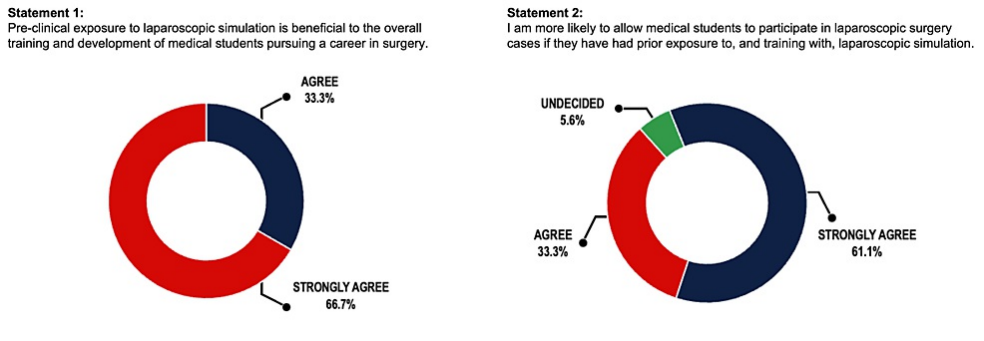
Survey results

An on-site survey was conducted among 18 surgeons consisting of 14 full-time faculty attendings, two post-graduate year-five residents, and two post-graduate year-three residents, all practicing in academic medicine with experience overseeing the training of medical students. Survey results indicated majority agreement with Statements 1 and 2. In response to Statement 1, 33.3% of surgeons strongly agreed, and 66.7% agreed. In response to Statement 2, 61.1% of surgeons strongly agreed, 33.3% agreed, and 5.6% were undecided (Figure 2).

## Discussion

This study highlights the significance of laparoscopic simulation as an early preparation tool in equipping medical students with the necessary skills for a career in surgery and its positive influence on their performance and competence in surgical residency programs. Our study indicates that surgeons acknowledge the advantages of early exposure to laparoscopic simulation in the training and advancement of medical students. Additionally, early exposure to laparoscopic simulation increases the likelihood of student participation in laparoscopic cases during surgical clerkship, which can further enhance their skills and competence in this area.

Several studies have reported the effectiveness of laparoscopic simulation in improving technical skills among medical students and enhancing their medical education. One study demonstrated that medical students who received systematic training through laparoscopic simulators exhibited improved basic skills in laparoscopic surgery, which could be effectively translated to the operating room [9]. To evaluate the development of skills in medical students relative to surgical residents, a 2003 study found that laparoscopic simulation training led to significant improvement in technical skills in both groups, with the “best” scores of the combined groups attained by a medical student [10]. Similarly, a 2001 study showed that medical students demonstrated equally significant improvement in laparoscopic skill development compared to second- and third-year surgical residents following laparoscopic simulation exposure [11]. This suggests that providing laparoscopic simulators to medical students can be a practical approach to augment their surgical skills and improve their overall learning experience. The 2013 study by Galiñanes et al. suggested that providing medical students with a standardized orientation to basic laparoscopy, including hands-on training and multimedia instruction, can positively affect their experience during their surgical clerkship. The study found that 100% of students who received the combined hands-on training simulation and multimedia instruction felt it helped during their clerkship, and 92% felt it had a positive effect [12]. Therefore, offering medical students access to laparoscopic simulation may be a helpful tool in enhancing medical education and providing a medium for skill development.

A laparoscopic simulation is a valuable tool for improving the technical skills of medical students and enhancing their education during surgical clerkships. Early exposure offers students interested in surgery the opportunity to develop the basic skills necessary to stand out during their surgical clerkships. In addition, this training provides opportunities for skill advancement, case participation, and better preparation for surgical residency. Academic medical institutions must prioritize providing accessible laparoscopic simulation training to their students. The acquisition of essential skills and experience in laparoscopic procedures will undoubtedly enhance the performance of medical students during their surgical clerkships and ensure senior students are adequately equipped to handle the demands of surgical residency. The provision of such training will not only augment the educational experience of medical students but also significantly contribute to the development of proficient and accomplished surgeons. Therefore, including laparoscopic simulation training as part of the medical curriculum is crucial to producing highly-skilled medical professionals capable of delivering excellent surgical care.

It should be noted that the present study has some limitations, including the small sample size and the need for more objective measures of laparoscopic simulation training. Future studies with larger survey sample sizes and objective assessments of laparoscopic simulation training are needed to investigate surgeon perspectives further.

## Conclusions

In conclusion, our study provides evidence to support the inclusion of laparoscopic simulation training in undergraduate medical education to enhance medical students' basic surgical skills and improve their clinical experience. Further research could inform the development of effective laparoscopic simulation training programs that prepare medical students transitioning to surgical residency.
